# One-Step ^18^F-Labeling of Estradiol Derivative for PET Imaging of Breast Cancer

**DOI:** 10.1155/2018/5362329

**Published:** 2018-03-05

**Authors:** Hongbo Huang, Ke Li, Gaochao Lv, Guiqing Liu, Xueyu Zhao, Qingzhu Liu, Shanshan Wang, Xi Li, Ling Qiu, Jianguo Lin

**Affiliations:** Key Laboratory of Nuclear Medicine of Ministry of Health and Jiangsu Key Laboratory of Molecular Nuclear Medicine, Jiangsu Institute of Nuclear Medicine, Wuxi 214063, China

## Abstract

Positron emission tomography (PET) imaging is a useful method to evaluate in situ estrogen receptor (ER) status for the early diagnosis of breast cancer and optimization of the appropriate treatment strategy. The ^18^F-labeled estradiol derivative has been successfully used to clinically assess the ER level of breast cancer. In order to simplify the radiosynthesis process, one-step ^18^F-^19^F isotope exchange reaction was employed for the ^18^F-fluorination of the tracer of** [**^**18**^**F]AmBF**_**3**_**-TEG-ES**. The radiotracer was obtained with the radiochemical yield (RCY) of ~61% and the radiochemical purity (RCP) of >98% within 40 min. Cell uptake and blocking assays indicated that the tracer could selectively accumulate in the ER-positive human breast cancer cell lines MCF-7 and T47D. In vivo PET imaging on the MCF-7 tumor-bearing mice showed relatively high tumor uptake (1.4~2.3 %D/g) and tumor/muscle uptake ratio (4~6). These results indicated that the tracer is a promising PET imaging agent for ER-positive breast cancers.

## 1. Introduction

Breast cancer has become the most common malignancy in women and the incidence of breast cancer is increasing over the world [1]. One of the primary reasons that cause death from breast cancer may be due to the lack of effective early diagnosis method [1]. Therefore, development of novel effective early diagnosis methods is critical for the treatment and survival of patients with breast cancer. It is acknowledged that estrogen plays an important role in the initiation and progression of breast cancer [2, 3]. The stimulatory effect of estrogen is mediated by nuclear estrogen receptors (ERs) [4]. Therefore, the estrogen receptor can serve as an important predictive biomarker of breast cancers [5]. The understanding of ER level is also essential for prognosis and optimization of the treatment strategy. The ER-positive (ER^+^) tumors often respond to the hormonal therapy, whereas the ER-negative (ER^−^) tumors usually require the surgical and chemotherapeutic interventions [2, 6, 7].

Positron emission tomography (PET) as an efficient noninvasive imaging technology has been applied successfully in clinic to provide the possibility for assessing the entire lesion and highly sensitive images of cancer diseases. Fluorine-18 is the most widely used radioisotope because of its ideal nuclear properties, such as low positron energy (0.64 MeV) and suitable physical half-life (109.8 min). Some fluorine-18-labeled estradiol derivatives, such as 16*α*-[^18^F]fluoroestradiol ([^18^F]FES) and C3-7*α*-[^18^F]FES, have been studied in preclinical evaluation, and tumor uptakes in PET imaging studies show a good correlation with the ER expression levels [7, 8]. However, the preparation of these radiotracers always require multisteps, including at least one drying step and semi-prep high performance liquid chromatography (HPLC) purification step. This will increase the risk of radiosynthesis failure and reduce the radiochemical yield (RCY) of radiotracer [9]. The multistep procedures are also challenged by the relatively short half-life of the radioisotope fluorine-18 [10]. Therefore, simplification of the radiosynthesis process is essential for the development and application of PET tracers based on the estradiol derivatives.

Recently, Liu and coworkers have developed a one-step ^18^F-labeling method with high radiochemical yield and high specific activity [9]. The ^18^F-labeling can be realized by the simple ^18^F-^19^F isotope exchange reaction on an ammoniomethyl-trifluoroborate (AmBF_3_) group without further drying of fluoride-18 ion and HPLC purification. Compared with other multistep radiolabeling methods, the one-step ^18^F-labeling approach is much more practical for clinical applications. However, this method is still not used widely to prepare PET imaging agents [11] and even no studies using this method to develop tracers of estradiol derivatives were reported.

Encouraged by the previous studies [11], in the present work we attempt to develop a novel estradiol-based PET tracer for imaging the ER-positive breast cancers by using the one-step ^18^F-labeling method to simplify the radiosynthesis procedure. The new tracer** [**^**18**^**F]AmBF**_**3**_**-TEG-ES** was prepared by conjugation of AmBF_3_ and estradiol with a PEG_4_ spacer. This new tracer could be easily prepared through one-step ^18^F-labeling method and simple purification in a short time. The in vitro stability, cytotoxicity, lipophilicity, and tumor cell uptake of the radiotracer were also investigated systematically. In addition, in vivo micro-PET imaging of breast cancer-bearing mice was also carried out and the results indicated that the tracer** [**^**18**^**F]AmBF**_**3**_**-TEG-ES** would be a potential PET imaging agent for the diagnosis of estrogen-dependent tumors.

## 2. Materials and Methods

### 2.1. General

All the materials were purchased from Energy Chemical and were all of reagent grade or analytical grade without further purification. Flouride-18 was obtained from a medical cyclotron (HM7, Sumitomo Heavy Industries) through a bombardment of ^18^O-enriched water. Human breast cancer cell lines T47D and MCF-7 were purchased from the Cell Bank of Chinese Academy of Sciences (Shanghai, China) and cultured in the 1640 medium and *α*-MEM medium (Biological Industries, Israel) with 10% (v/v) fetal bovine serum (Biological Industries, Israel) in a 37°C incubator under 5% CO_2_, respectively. A 1470 Wizard *γ* counter (Perkin-Elmer Corporation, USA) was employed in the studies of cellular uptake.

The semi-prep HPLC (Waters, USA) with a Waters 2998 photodiode array detector (PDA) and C18 HPLC column (5 *μ*m, 250 × 19 mm, Phenomenex) was employed for purification of compounds. The flow rate of semi-prep HPLC was 3 mL/min. The semi-prep HPLC method for the purification of** AmBF**_**3**_**-TEG-ES** has been listed in Table 1.

The analytical HPLC equipped with a C18 column (5 *μ*m, 250 × 4.6 mm, Phenomenex) and a Waters 2487 dual *λ* absorbance detector was used for purity identification of the precursor on a Waters Breeze system. A Radiomatic 610TR flow scintillation analyzer (Perkin-Elmer) was necessary for the quality control analysis of radiolabeled compounds in this system. The flow rate of analytical HPLC was 1 mL/min. The analytical HPLC method for quality control has been listed in Table 2.

*Animals*. BALB/c nude mice (18–20 g; 4–6 weeks old; SLAC Laboratory Animal Co. Ltd., Shanghai, China) were used for animal experiments. Mice were housed with free access to food and water and allowed ample time to acclimatize before the experiments. The tumor-bearing mice were established by subcutaneous injection of MCF-7 cells (5 × 10^6^) suspended in PBS (100 *μ*L) in the right shoulder of each nude mouse. The tumors were allowed to grow for around 3-4 weeks to reach the size of 0.5–1.0 cm in diameter for in vivo studies. All procedures and animal protocols were approved by the Animal Care and Ethnics Committee of Jiangsu Institute of Nuclear Medicine.

Nuclear magnetic resonance spectrometers (^1^H-NMR, ^13^C-NMR, and ^19^F-NMR, Bruker DRX-400, Bruker, Germany) were used to obtain spectra of samples dissolved in* d*_6_*-*DMSO, and the chemical shifts were referenced to tetramethylsilane (TMS). Electrospray ionization mass spectrometry (ESI-MS) was obtained on a Waters Platform ZMD4000 quadrupole tandem mass spectrometer. A Perkin-Elmer 240C Elemental Analyzer was employed for the elemental analysis (C, H, and N). FT-IR spectra of the cold compound** AmBF**_**3**_**-TEG-ES** was obtained using a FT-IR spectrometer (TENSOR27, Bruker) in the range of 400–4000 cm^−1^.

### 2.2. Chemical Synthesis

#### 2.2.1. Synthesis of Compound** 1**

Compound** 1** was synthesized according to the method reported previously [12]. Tetraethylene glycol (4.51 g, 23 mmol) was first dissolved in THF (20 mL) at room temperature, and then KOH (2.06 g, 36 mmol) dissolved in H_2_O (3 mL) was added to the solution. Subsequently,* p*-toluenesulfonyl chloride (4.37 g, 23 mmol) was dissolved in THF and added drop-wise to the solution. The reaction mixture was stirred under ice-bath. The reaction solution was diluted with NaCl saturated solution (50 mL) and extracted with CH_2_Cl_2_ (3 × 50 mL). The combined organic layer was dried over anhydrous sodium sulfate and the solvent was evaporated under reduced pressure. Then, the crude product was purified by column chromatography using CH_2_Cl_2_/CH_3_OH (30 : 1 v/v) to obtain the compound** 1**. Yield: 61%, 5.23 g. ESI-MS: m/z 349.23 [M + H]^+^.

#### 2.2.2. Synthesis of Compound** 2**

Compound** 1 **(5.23 g, 15 mmol) and NaN_3_ (2.90 g, 45 mmol) were dissolved in CH_3_CN (20 mL) and refluxed at 95°C for 16 h. Then the solution of the reaction mixture was cooled down to room temperature and NaCl saturated solution (50 mL) was added. After being extracted with CH_2_Cl_2_, the combined organic layer was dried with anhydrous sodium sulfate and the solvent was evaporated under reduced pressure to obtain the compound** 2**. Yield: 89%, 2.93 g. ESI-MS: m/z 242.22 [M + Na]^+^.

#### 2.2.3. Synthesis of Compound** 3**

Compound** 2 **(2.93 g, 13 mmol) was dissolved in CH_2_Cl_2_ (20 mL), and then DMAP (0.35 g, 2.9 mmol) and TEA (8.9 mL, 67 mmol) were added. When the reaction mixture was stirred under ice-bath,* p*-toluenesulfonyl chloride (2.47 g, 13 mmol) in CH_2_Cl_2_ was added drop-wise to the solution. Then the reaction mixture was stirred at room temperature overnight. Subsequently, the solution of reaction mixture was washed with HCl aqueous solution (1 M, 3 × 50 mL) and NaCl saturated solution (3 × 50 mL). Then, the organic layer was dried with anhydrous sodium sulfate and the solvent was evaporated under reduced pressure. The crude product was purified by flash column chromatography hexane/acetic ether (2 : 1 v/v) to provide compound** 3**. Yield: 66%, 3.20 g. ESI-MS: m/z 396.25 [M + Na]^+^.

#### 2.2.4. Synthesis of Compound** 4**

The suspension of compound** 3** (3.20 g, 8.6 mmol), N,N-dimethylethanolamine (772 *μ*L, 7.5 mmol), and KOH (1.62 g, 29 mmol) in THF (50 mL) was refluxed at 70°C for 12 h. The reaction mixture was filtered and the filtrate was collected. The solvent was evaporated under reduced pressure and then H_2_O (50 mL) was added and extracted with CH_2_Cl_2_ (3 × 50 mL). The combined organic layer was dried over hydrous sodium sulfate and then evaporated under reduced pressure. The crude product was purified through flash column chromatography CH_2_Cl_2_/CH_3_OH (10 : 1 v/v). Yield: 45%, 1.12 g. ESI-MS: m/z 291.31 [M + H]^+^.

#### 2.2.5. Synthesis of Compound** 5**

Compound** 4** (480 mg, 1.6 mmol) was dissolved in anhydrous THF (10 mL). 2-(Bromomethyl)-4,4,5,5-teramethyl-1,3,2-dioxaboralane (900 *μ*L, 4.92 mmol) was added drop-wise to the above solution at room temperature under nitrogen atmosphere. Then the reaction mixture was stirred for 3 h. The solvent was evaporated under reduced pressure to obtain compound** 5**. Compound** 5 **was used without any purification. Yield: 67%, 478 mg. ESI-MS: m/z 431.49 [M]^+^.

#### 2.2.6. Synthesis of Compound** 6**

Compound** 5** (478 mg, 1.1 mmol) was dissolved in DMF (1 mL). KHF_2_ (3 M, 1 mL) and HCl (4 M, 1 mL) were added to the above solution. The reaction was stirred at 45°C for 2 h. Then the reaction was quenched by NH_4_OH (20 *μ*L). Crude product was purified through silica gel CH_2_Cl_2_/CH_3_OH (20 : 1 v/v) to give compound** 6**. Yield: 16%, 66 mg. ESI-MS: m/z 395.34 [M + Na]^+^.

#### 2.2.7. Synthesis of Compound** 7 (AmBF**_**3**_**-TEG-ES)**

Click chemistry was used for the synthesis of compound** 7 (AmBF**_**3**_**-TEG-ES)**. Briefly, compound** 6** (66 mg, 0.18 mmol), ethinylestradiol (64 mg, 0.22 mmol), tris(2-benzimidazolylmethyl)amine (7.2 mg, 0.018 mmol), sodium ascorbate (71 mg, 0.36 mmol), and CuSO_4_·5H_2_O (9.1 mg, 0.036 mmol) were reacted in 4 mL DMF/H_2_O (1 : 1) at 45°C for 1 h. Then, semi-prep HPLC was used for the purification of the crude product (Table 1). Then it was lyophilized to give the final product** 7** (**AmBF**_**3**_**-TEG-ES**). The compound** AmBF**_**3**_**-TEG-ES** was analyzed by an analytical HPLC (Table 2). Yield: 73%, 88 mg. ESI-MS: m/z 691.57 [M + Na]^+^. ^1^H-NMR (*d*_6_*-*DMSO, 400 MHz, *δ*: ppm): *δ* 7.84 (s, 1H), 6.96 (d, 1H,* J *= 8.0 Hz), 6.48 (d, 1H,* J *= 12.0 Hz), 6.42 (s, 1H), 4.50 (t, 2H,* J *= 4.0 Hz), 3.79~3.83 (m, 4H), 3.42~3.52 (m, 14H), 3.00 (s, 6H), 2.70 (t, 2H,* J *= 4.0 Hz), 2.35 (m, 3H), 1.24~2.09 (m, 12H), 0.93 (s, 3H); ^13^C-NMR (*d*_6_*-*DMSO, 101 MHz, *δ*: ppm): *δ* 155.3, 154.3, 137.6, 130.9, 126.4, 123.5, 115.4, 113.1, 81.5, 70.3, 70.2, 70.1, 70.0, 69.9, 69.9, 69.3, 65.1, 64.9, 53.8, 49.7, 48.0, 47.2, 43.7, 37.6, 33.1, 29.7, 29.5, 27.7, 26.5, 24.0, 14.8; ^19^F-NMR (*d*_6_*-*DMSO, 376 MHz, *δ*: ppm): *δ*  −135.5. Anal. Elem. Anal. Calc. for C_33_H_52_BF_3_N_4_O_6_: C, 59.28; H, 7.84; N, 8.38. Found: C, 59.77; H, 7.63; N, 8.48. IR (KBr): 3391 (w), 2930 (m), 1611 (m), 1454 (s), 1352 (m), 971 (m).

### 2.3. Stability of** AmBF**_**3**_**-TEG-ES**

The stability of the cold compound** AmBF**_**3**_**-TEG-ES** was studied in pyridazine-HCl buffer (1.0 M, pH 2.0–2.5) at different temperatures. Briefly,** AmBF**_**3**_**-TEG-ES** was dissolved in pyridazine-HCl buffer and then incubated at various temperature (50, 60, 70, 80, 90, or 100°C) for 30 min, respectively. After incubation, a little sample was taken out for stability assay using HPLC analysis (Table 1).

### 2.4. Cytotoxicity Assay of** AmBF**_**3**_**-TEG-ES**

Traditional 3-(4,5-dimethyl-thiazol-2-yl)-2,5-diphenyltetrazolium bromide (MTT) assay was used to assess the cytotoxicity of** AmBF**_**3**_**-TEG-ES** against ER^+^ human breast cancer cell lines T47D and MCF-7. Briefly, the cells were seeded into 96-well plates with 100 *μ*L medium at a density of 5 × 10^3^ cells per well. Then the cells were incubated in a 37°C incubator with 5% CO_2_ for 12 h. Subsequently, the medium in each well was replaced by different concentrations (12.5, 25, 50, and 100 *μ*M) of the cold compound** AmBF**_**3**_**-TEG-ES **(200 *μ*L/well), which were diluted with the culture medium. Then, the plates were incubated for 3, 6, 12, and 24 h, respectively. After treatment with** AmBF**_**3**_**-TEG-ES**, MTT solution (20 *μ*L, 5 mg/mL) was added to each well and all the plates were incubated for another 4 h. After that, medium in each well was removed and replaced by DMSO (150 *μ*L). After the culture plates were shaken for 10 min, the optical density (OD) values were measured on a microplate reader (BioTek Instruments, Inc. Vermont, USA) at 490 nm. All tests were carried out in 6 repeats for at least three independent experiments.

### 2.5. Radiosynthesis of** [**^**18**^**F]AmBF**_**3**_**-TEG-ES**

Fluoride-18 (100–300 mCi) produced by cyclotron was trapped on an anion-exchange resin and then eluted with pyridazine-HCl buffer (200 *μ*L) into the reaction polypropylene tube. The precursor** AmBF**_**3**_**-TEG-ES** (10 *μ*L, 25 mM in pyridazine-HCl buffer) was added to the tube. Then the mixture was heated at different temperature for 30 min, respectively. After reaction, the reaction mixture was loaded on a C18 light cartridge when diluted with water (10 mL). Then, the C18 light cartridge was washed with water (3 × 10 mL). The pure tracer** [**^**18**^**F]AmBF**_**3**_**-TEG-ES** was obtained after being eluted with ethanol/saline (0.5 mL) into a glass vial. The target product** [**^**18**^**F]AmBF**_**3**_**-TEG-ES **should be diluted with saline for further biological evaluation. A small sample was taken out for radio-HPLC analysis using the condition listed in Table 2.

### 2.6. Determination of Octanol-Water Partition Coefficient

Octanol (50 mL) and distilled water (50 mL, pH 7.0) were mixed by oscillation at 25°C for 24 h. After standing, a separatory funnel was used to separate the two layers. The tracer (30 *μ*Ci) in distilled water (500 *μ*L) was added to a polypropylene tube. Octanol (500 *μ*L) was also added to the tube and then oscillated on a vortex mixer for 5 min. Next, the tube was centrifuged for 5 min at 4000 g to separate the two layers. In each layer, the radioactivity of samples (100 *μ*L) in triplicate was measured by a *γ*-counter. The partition coefficient (log⁡*P*) value was calculated using the equation log⁡*P* = log⁡(*C*_*o*_/*C*_*w*_), where *C*_*o*_ is the radioactivity of the tracer in the n-octanol layer and *C*_*w*_ is the radioactivity in the water layer. The result was the average of three independent experiments.

### 2.7. Cell Uptake and Block Studies of** [**^**18**^**F]AmBF**_**3**_**-TEG-ES**

The cell uptake and block studies of the radiotracer** [**^**18**^**F]AmBF**_**3**_**-TEG-ES **were conducted on human breast cancer cell lines MCF-7 and T47D, respectively. Firstly, MCF-7 or T47D cells (500 *μ*L/well, 4 × 10^5^ cells/mL) were seeded in 24-well culture plates and incubated overnight. Then the medium was replaced by fresh serum-free medium (500 *μ*L) containing** [**^**18**^**F]AmBF**_**3**_**-TEG-ES **(0.5 *μ*Ci). The plates were incubated in a 37°C incubator for 15, 30, 60, and 120 min, respectively. For the block study, the cells were pretreated with excess of estradiol for 30 min. At each time point, the supernatant in each well was removed and then the cells were washed with ice-cold PBS (500 *μ*L/well) twice. Next, NaOH solution (0.1 M) was added to lyse the cells. The cell lysates were collected and the following eluents of PBS were combined. The radioactivity of cell lysates was determined using a *γ*-counter. The cell uptake rate was expressed as the percentage of the total added radioactive dose (%AD).

### 2.8. PET Imaging

Small animal PET imaging was performed on an Inveon micro-PET scanner (Siemens Medical Solutions, Germany). Imaging studies were carried out on female Balb/c nude mice bearing the human breast cancer cell line MCF-7. The mice were anesthetized with 1.5%–2% isoflurane, positioned prone, immobilized, injected with** [**^**18**^**F]AmBF**_**3**_**-TEG-ES** (150 *μ*Ci) via the tail vein, and imaged dynamically for the first hour. Then, static scanning images were collected immediately at 0.5, 1, and 2 h after injection. The obtained images were reconstructed using 3D ordered subset expectation maximization (OSEM 3D/SP-MAP) and then processed using the Siemens Inveon Research Workplace (IRW2.0.0.1050). Regions of interest (ROIs) were drawn over tumors and main organs, and average signal levels in the ROIs were measured. The %ID/g was also calculated.

## 3. Results and Discussion

### 3.1. Design and Synthesis of** AmBF**_**3**_**-TEG-ES**

The harsh radiosynthesis conditions and purification procedure limit the clinical applications of the estradiol-based PET imaging agents. Thus, development of new radiolabeling method is imperative. In our previous work, a novel PET tracer** [**^**18**^**F]AmBF**_**3**_**-ES** was reported, which was synthesized by conjugating the estradiol with the AmBF_3_ directly [13]. Excitedly, the radiotracer** [**^**18**^**F]AmBF**_**3**_**-ES** was obtained in high yield within 30 min obviating HPLC purification through one-step ^18^F-labeling approach, and it also showed high serum stability and cellular uptakes. However, the following in vivo tumor imaging studies were disappointing as the tumor tissues showed no uptakes. In order to further develop the simple one-step ^18^F-labeling method and improve the in vivo imaging effect of ^18^F-labeled estradiol derivatives, the new tracer** [**^**18**^**F]AmBF**_**3**_**-TEG-ES** was designed and prepared by conjugation of AmBF_3_ and estradiol with a PEG_4_ spacer. Notably, the embedding of PEG_4_ spacer between AmBF_3_ and estradiol was to adjust the solubility of the tracer in saline, to reduce the steric hindrance for the estradiol-estrogen receptor binding, and to improve the in vivo pharmacokinetics of the tracer** [**^**18**^**F]AmBF**_**3**_**-TEG-ES**, which might overcome the disadvantages of the tracer** [**^**18**^**F]AmBF**_**3**_**-ES** reported previously.

**Scheme 1:**
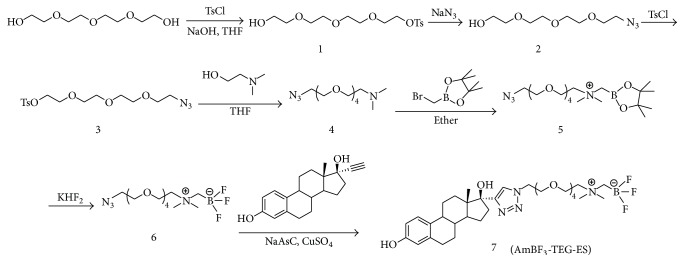
Synthesis route of** AmBF**_**3**_**-TEG-ES**.

**Figure 1:**
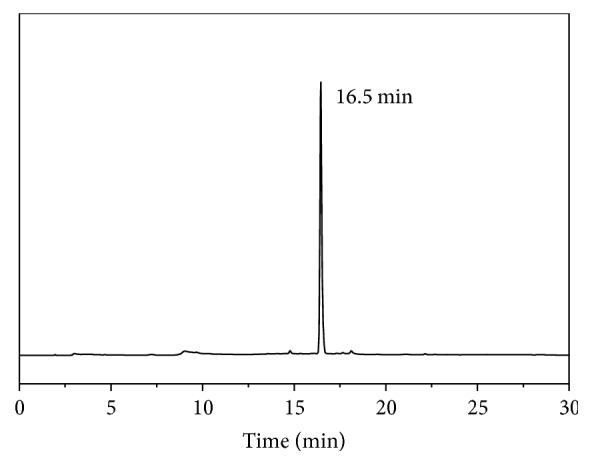
HPLC chromatogram of the precursor** AmBF**_**3**_**-TEG-ES**.

The synthetic route of** AmBF**_**3**_**-TEG-ES** has been shown in Scheme 1. Compound** 3** was obtained with high yield by three steps from tetraethylene glycol, which further reacted with N,N′-dimethylethanolamine to generate compound** 4**. And then compound** 6** was prepared according to the method by Liu et al. [9, 14]. Finally,** AmBF**_**3**_**-TEG-ES** was obtained through a click reaction of compound** 6** and ethinylestradiol. Then the precursor was purified on a semi-prep HPLC with a high yield of 73%. The purity of** AmBF**_**3**_**-TEG-ES **was confirmed by HPLC analysis. As shown in Figure 1, the single peak recorded at 280 nm demonstrates high chemical purity of the precursor. All the intermediates were characterized by ESI-MS, and the corresponding molecular ion peaks ([M + H]^+^ or [M + Na]^+^) indicated the successful synthesis of the compounds. As shown in Figure S1, the peaks 669.5 and 691.5 were assigned to the molecular ion peaks [M + H]^+^ and [M + Na]^+^ of the cold compound** AmBF**_**3**_**-TEG-ES**, respectively. Then ^1^H-, ^13^C-, and ^19^F-NMR spectra further confirmed the structure of** AmBF**_**3**_**-TEG-ES**. The peaks at *δ* 6.42 ppm (hydrogen atom of triazole) of ^1^H-NMR spectrum and those at *δ* 126.4 and 123.5 ppm (carbon atoms of triazole) of ^13^C-NMR spectrum as well as the disappearance of alkynyl peaks in the ^1^H-NMR and ^13^C-NMR spectra demonstrated the successful occurrence of the click reaction (Figures S2 and S3). The peak at −75.7 ppm of ^19^F-NMR spectrum was assigned to TFA in the final compound and the single peak at −135.5 ppm also indicated the purity of the compound** AmBF**_**3**_**-TEG-ES **(Figure S4). All of these results indicated that the compound** AmBF**_**3**_**-TEG-ES **was successfully synthesized with a high chemical purity.

### 3.2. Stability of** AmBF**_**3**_**-TEG-ES**

**Figure 2:**
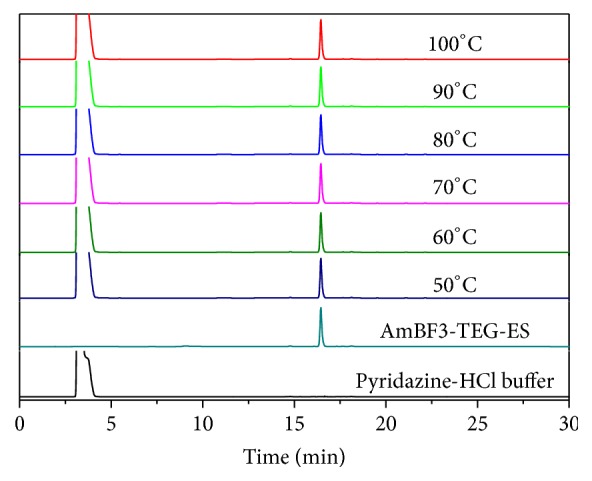
Stability of** AmBF**_**3**_**-TEG-ES** in pyridazine-HCl buffer at various temperatures.

Since the one-step ^18^F-^19^F isotope exchange labeling reaction was performed in the pyridazine-HCl buffer (pH = 2.0–2.5) around 80°C, the stability of** AmBF**_**3**_**-TEG-ES** in the pyridazine-HCl buffer was studied by HPLC analysis at the temperature range of 50–100°C. As shown in Figure 2, the peak at 3.35 min corresponded to the buffer while** AmBF**_**3**_**-TEG-ES** showed a single peak at 16.5 min. After incubation at different temperatures, the HPLC analysis of **AmBF**_**3**_-**TEG-ES** in pyridazine-HCl buffer showed a single peak at 16.5 min. This indicated that** AmBF**_**3**_**-TEG-ES** was stable enough in the pyridazine-HCl buffer after incubation even up to 100°C for 30 min and the ^18^F-labeling reaction can be carried out in the pyridazine-HCl buffer.

### 3.3. Cytotoxicity of** AmBF**_**3**_**-TEG-ES**

**Figure 3:**
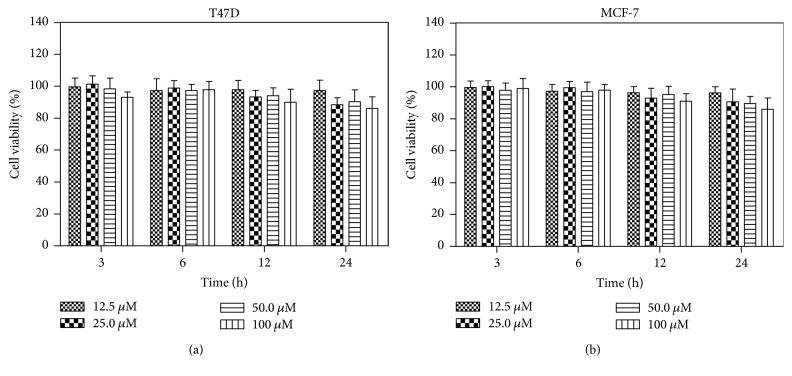
Cytotoxicity of** AmBF**_**3**_**-TEG-ES** against breast cancer cells T47D (a) and MCF-7 (b).

The good biocompatibility is essential for the application of a PET imaging agent [15]. Therefore, the cytotoxicity of** AmBF**_**3**_**-TEG-ES** against the human breast cancer cells T47D and MCF-7 was assessed by MTT assay. As can be seen from Figures 3(a) and 3(b), the cell viability of T47D and MCF-7 did not change significantly after incubation for different time with** AmBF**_**3**_**-TEG-ES** at the concentration ranging from 12.5 to 100 *μ*M. In fact, more than 87% of both cells still survived after treatment with** AmBF**_**3**_**-TEG-ES** at the high concentration of 100 *μ*M for 24 h. Hence, it is inferred that** AmBF**_**3**_**-TEG-ES** possesses negligible cytotoxicity and good biocompatibility. Compared with the compound** AmBF**_**3**_**-ES** reported by our group previously [13], the cytotoxicity of** AmBF**_**3**_**-TEG-ES** against MCF-7 and T47D cells showed no significant difference, indicating that the short PEG linker had almost no influence on the cytotoxicity of** AmBF**_**3**_**-TEG-ES**.

### 3.4. Radiosynthesis of** [**^**18**^**F]AmBF**_**3**_**-TEG-ES**

**Figure 4:**
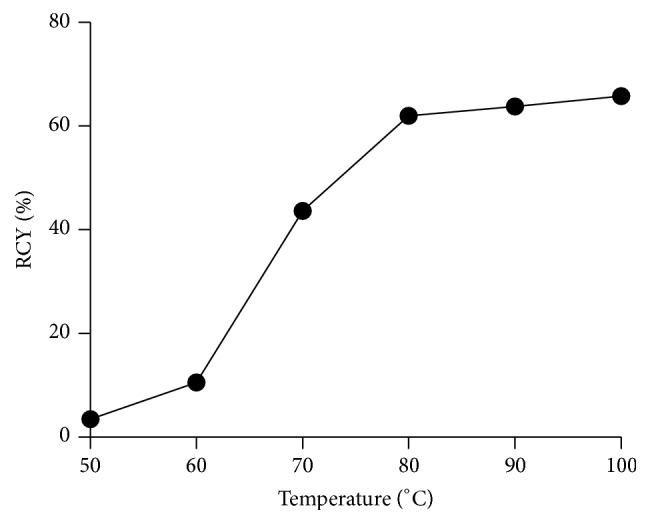
Radiochemical yield (RCY) of** [**^**18**^**F]AmBF**_**3**_**-TEG-ES** in pyridazine-HCl buffer (1.0 M, pH 2.0–2.5) at different incubation temperature.

**Figure 5:**
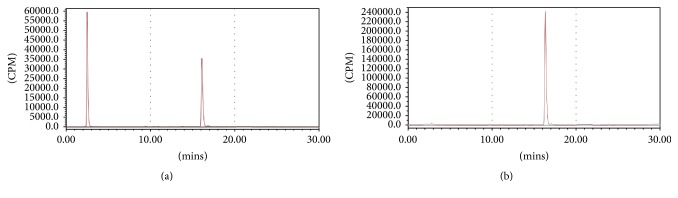
Radio-HPLC analysis of** [**^**18**^**F]AmBF**_**3**_**-TEG-ES** (*t*_*R*_ = 16.3 min) before purification (a) and after C18 purification (b).

^18^F-labeling of the precursor** AmBF**_**3**_**-TEG-ES** was performed using the one-step ^18^F-^19^F isotope exchange approach. To optimize the condition of ^18^F-labeling, the precursor** AmBF**_**3**_**-TEG-ES** was heated with ^18^F-fluoride in the pyridazine-HCl buffer (pH = 2.0–2.5) at different temperatures. As shown in Figure 4, the RCYs of** [**^**18**^**F]AmBF**_**3**_**-TEG-ES** increased significantly with the increasing temperature within 30 min. Especially at 80°C, the RCY reached 61% and the RCP was over 98% after C18 purification (Figure 5), but further increasing the reaction temperature did not produce a higher RCY. Therefore, the optimum radiolabeling condition (80°C, 30 min) was selected for further experiments. It was worth mentioning that the radiosynthesis process of the ER imaging agent was significantly simplified in comparison with that of ^18^F-FES [16–18]. For the radiosynthesis of the widely studied ER imaging agent ^18^F-FES, two reaction steps and semi-prep were required which resulted in a long synthesis time [16–18]. However, ^18^F-labeling of the ER imaging agent through the one-step ^18^F-^19^F isotope exchange approach remarkably shortened the synthesis time and improved the radiolabeling efficiency, which obviated drying of fluoride-18 ion and HPLC purification with the total radiosynthesis time of 40 min. Moreover, the C18 column instead of HPLC purification also led to a high RCY with a satisfactory RCP. All the results indicated that the one-step ^18^F-^19^F isotope exchange could offer a more convenient method for the ^18^F-labeling process and it was more suitable for clinical application.

**Figure 6:**
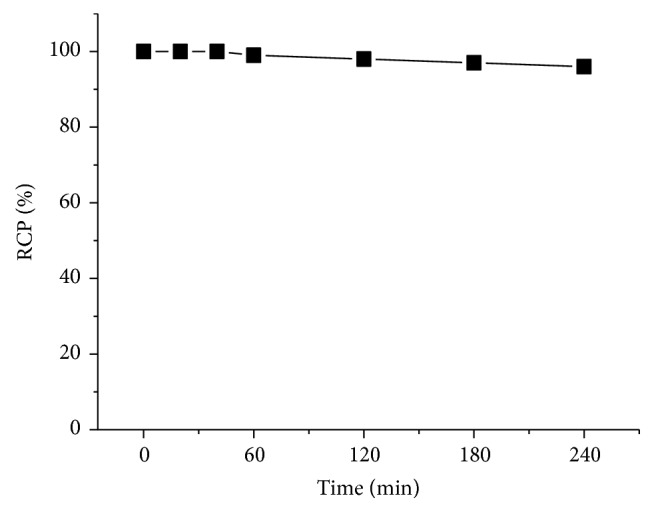
In vitro stability test of** [**^**18**^**F]AmBF**_**3**_**-TEG-ES** after incubation in PBS at 37°C for different periods of time.

In order to evaluate the stability of** [**^**18**^**F]AmBF**_**3**_**-TEG-ES **in vitro,** [**^**18**^**F]AmBF**_**3**_**-TEG-ES **was incubated in PBS (pH = 7.4) for 4 h at 37°C. As shown in Figure 6, the probe showed high stability (>95%) in PBS for up to 4 h. The high stability of** [**^**18**^**F]AmBF**_**3**_**-TEG-ES **indicated that this probe was suitable for further biological studies.

### 3.5. Octanol-Water Partition Coefficient

The octanol-water partition coefficient (log⁡*P*) of a compound always reflects its lipid solubility and correlates with its pharmacokinetics, such as cell membrane permeability and tissue distribution, according to the studies of quantitative structure-activity relationship [19]. The log⁡*P* value for** [**^**18**^**F]AmBF**_**3**_**-TEG-ES** was determined to be −0.17 ± 0.03, which displayed better hydrophilicity than** [**^**18**^**F]AmBF**_**3**_**-ES** (0.52 ± 0.09) as expected. The lipophilicity of** [**^**18**^**F]AmBF**_**3**_**-TEG-ES **may endow it with suitable pharmacokinetics in vivo for tumor imaging. The hydrophilicity of** [**^**18**^**F]AmBF**_**3**_**-TEG-ES **may endow it with a suitable pharmacokinetics in vivo for tumor imaging.

### 3.6. Cell Uptake and Blocking Assay of** [**^**18**^**F]AmBF**_**3**_**-TEG-ES**

**Figure 7:**
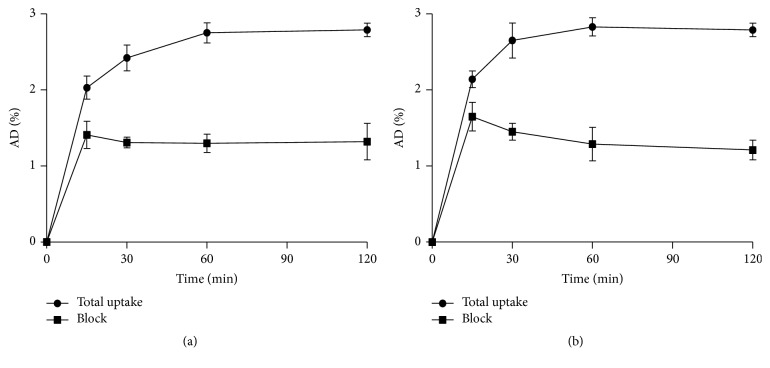
Cell uptake and block studies of** [**^**18**^**F]AmBF**_**3**_**-TEG-ES** in MCF-7 (a) and T47D cells (b).

Since estrogen receptor is overexpressed in human breast cancer cell lines MCF-7 and T47D, cell uptake and block studies of** [**^**18**^**F]AmBF**_**3**_**-TEG-ES** were performed on these two kinds of cells, respectively. As shown in Figure 7(a), the cell uptake rate of** [**^**18**^**F]AmBF**_**3**_**-TEG-ES** approached a plateau of ~2.7% after incubation at 37°C for 60 min and remained at a similar uptake level to 120 min, indicating that the radiotracer could bind quickly to the ER^+^ MCF-7 cells. Similarly, approximately 2.8% cell uptake of** [**^**18**^**F]AmBF**_**3**_**-TEG-ES** in T47D cells was determined at 60 min and almost no change up to 120 min, which was consistent with that of MCF-7 cell lines (Figure 7(b)). For the blocking study, the cell uptake rate in MCF-7 cells decreased from 2.7% to 1.3% at presence of excess estradiol at 60 min, which decreased about 52% compared with the total cell uptake. In T47D cells, the cell uptake rate was only 1.2% at presence of excess estradiol at 60 min. These data showed that the cell uptake in ER^+^ breast cancer cells could be effectively blocked by estradiol and further demonstrated the specificity of** [**^**18**^**F]AmBF**_**3**_**-TEG-ES** to ER.

### 3.7. Micro-PET Imaging

**Figure 8:**
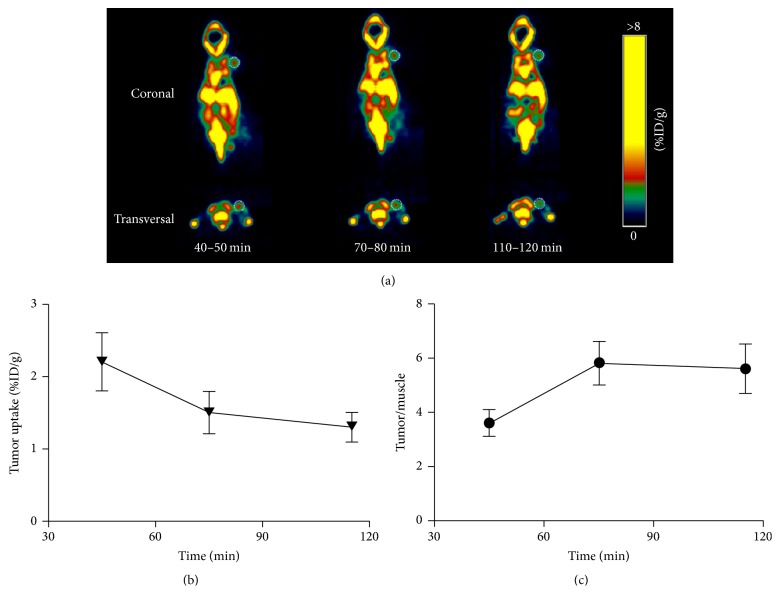
(a) Micro-PET imaging of** [**^**18**^**F]AmBF**_**3**_**-TEG-ES **in MCF-7 tumor-bearing nude mice. (b) Tumor uptake at different time. (c) Tumor/muscle uptake ratio at different time.

In vivo micro-PET imaging was also carried out to investigate the specificity of** [**^**18**^**F]AmBF**_**3**_**-TEG-ES** to localize in ER-positive tumor. The MCF-7 tumor-bearing nude mice were used for imaging at different time periods varying from 40 to 120 min. As shown in Figures 8(a) and 8(b),** [**^**18**^**F]AmBF**_**3**_**-TEG-ES **accumulated in ER^+^ MCF-7 tumors with an uptake of 1.4 to 2.3 %D/g, showing visualization of the ER-positive tumors. The highest uptake in tumor was observed at 40–50 min after injection, and then the uptake decreased slightly with the time prolonging. However, the tumor/muscle ratio increased with the increase of time, which was about 4 at the initial time point of 40 min (Figure 8(c)) and then increased to about 6 at 70 min and remained 6 at 110 min. Noteworthy is that the previously reported compound** [**^**18**^**F]AmBF**_**3**_**-ES **showed no uptake in the same tumor tissues, but the compound** [**^**18**^**F]AmBF**_**3**_**-TEG-ES **showed an improvement, which may be attributed to the introduction of the PEG spacer [20]. Hence, the results of micro-PET imaging further indicate that** [**^**18**^**F]AmBF**_**3**_**-TEG-ES **has potential application for the ER-positive tumor imaging.

## 4. Conclusions

In summary, a novel estradiol-based PET imaging agent** [**^**18**^**F]AmBF**_**3**_**-TEG-ES** was synthesized and characterized, and its in vitro and in vivo biological performance was also evaluated. The radiosynthesis of this compound was quite applicable in clinic by using the one-step ^18^F-^19^F isotope exchange reaction within 40 min, which obviated the drying of fluoride-18 ion and the HPLC purification. The high RCY, excellent RCP, good biocompatibility, high stability, and specific ER-targeting ability suggested that the radiotracer** [**^**18**^**F]AmBF3-TEG-ES** might be a promising PET imaging agent for diagnosis of ER^+^ breast cancers.

## Figures and Tables

**Table 1:** The gradient elution condition for semi-prep HPLC analysis of **AmBF**_**3**_**-TEG-ES.**

Time (min)	Flow (mL/min)	% A	% B
0.01	3.00	80.0	20.0
3.00	3.00	80.0	20.0
15.00	3.00	65.0	35.0
20.00	3.00	65.0	35.0
25.00	3.00	50.0	50.0
30.00	3.00	30.0	70.0
35.00	3.00	10.0	90.0
40.00	3.00	80.0	20.0

**Table 2:** The gradient elution condition of analytical HPLC.

Time (min)	Flow	% A	% B
0.01	1.00	80.0	20.0
3.00	1.00	80.0	20.0
25.00	1.00	30.0	70.0
30.00	1.00	80.0	20.0

## Supplementary Materials

Supplementary Materials Supplementary date available: ESI-MS and NMR spectra. Figure S1: ESI-MS of compound **A****m****B****F**_3_-**T****E****G**-**E****S**. Figure S2: ^1^H-NMR of compound **A****m****B****F**_3_-**T****E****G**-**E****S**. Figure S3: ^13^C-NMR of compound **A****m****B****F**_3_-**T****E****G**-**E****S**. Figure S4: ^19^F-NMR of compound **A****m****B****F**_3_-**T****E****G**-**E****S**.

## References

[1] Torre L. A., Bray F., Siegel R. L., Ferlay J., Lortet-Tieulent J. (2015). Global cancer statistics, 2012. *CA: A Cancer Journal for Clinicians*.

[2] Chauhan K., Arun A., Singh S. (2016). Bivalent Approach for Homodimeric Estradiol Based Ligand: Synthesis and Evaluation for Targeted Theranosis of ER(+) Breast Carcinomas. *Bioconjugate Chemistry*.

[3] Bharti J. N., Rani P., Kamal V., Agarwal P. N. (2015). Angiogenesis in breast cancer and its correlation with estrogen, Progesterone receptors and other prognostic factors. *Journal of Clinical and Diagnostic Research*.

[4] Linden H. M., Dehdashti F. (2013). Novel methods and tracers for breast cancer imaging. *Seminars in Nuclear Medicine*.

[5] Tecalco-Cruz A. C., Pérez-Alvarado I. A., Ramírez-Jarquín J. O., Rocha-Zavaleta L. (2017). Nucleo-cytoplasmic transport of estrogen receptor alpha in breast cancer cells. *Cellular Signalling*.

[6] Losurdo A., Rota S., Gullo G. (2017). Controversies in clinicopathological characteristics and treatment strategies of male breast cancer: A review of the literature. *Critical Review in Oncology/Hematology*.

[7] Xia X., Feng H., Li C., Qin C., Song Y., Zhang Y. (2016). 99mTc-labeled estradiol as an estrogen receptor probe: Preparation and preclinical evaluation. *Nuclear Medicine and Biology*.

[8] Kue C. S., Kamkaew A., Burgess K., Kiew L. V., Chung L. Y., Lee H. B. (2016). Small Molecules for Active Targeting in Cancer. *Medicinal Research Reviews*.

[9] Liu Z., Lin K.-S., Bénard F. (2015). One-step 18 F labeling of biomolecules using organotrifluoroborates. *Nature Protocols*.

[10] Liu Z., Pourghiasian M., Radtke M. A. (2014). An organotrifluoroborate for broadly applicable one-step18F-labeling. *Angewandte Chemie International Edition*.

[11] Lin J., Wang W., Li K. (2017). Development of a kit-like radiofluorinated biomolecule leading to a controlled self-assembly of 18F nanoparticles for a smart PET imaging application. *Chemical Communications*.

[12] Shen B., Jeon J., Palner M. (2013). Positron emission tomography imaging of drug-induced tumor apoptosis with a caspase-triggered nanoaggregation probe. *Angewandte Chemie International Edition*.

[13] Liu G., Wang W., Lin J., Li K., Lv G., Zhao X. (2017). Kit-like 18F-labeling of an estradiol derivative as a potential PET imaging agent for estrogen receptor-positive breast cancer. *Journal of Radioanalytical and Nuclear Chemistry*.

[14] Liu Z., Li Y., Lozada J. (2013). Kit-like 18F-labeling of RGD-19F-Arytrifluroborate in high yield and at extraordinarily high specific activity with preliminary in vivo tumor imaging. *Nuclear Medicine and Biology*.

[15] An F. F., Kommidi H., Chen N., Ting R. (2017). A conjugate of pentamethine cyanine and18F as a positron emission tomography/near-infrared fluorescence probe for multimodality tumor imaging. *International Journal of Molecular Sciences*.

[16] Yoo J., Dence C. S., Sharp T. L., Katzenellenbogen J. A., Welch M. J. (2005). Synthesis of an estrogen receptor *β*-selective radioligand: 5-[ 18F]fluoro-(2R∗,3S∗)-2,3-bis(4-hydroxyphenyl) pentanenitrile and comparison of in vivo distribution with 16*α*-[ 18F]fluoro-17*β*-estradiol. *Journal of Medicinal Chemistry*.

[17] Zhao Z., Yoshida Y., Kurokawa T., Kiyono Y., Mori T., Okazawa H. (2013). 18F-FES and 18F-FDG PET for differential diagnosis and quantitative evaluation of mesenchymal uterine tumors: Correlation with immunohistochemical analysis. *Journal of Nuclear Medicine*.

[18] Mori T., Kasamatsu S., Mosdzianowski C., Welch M. J., Yonekura Y., Fujibayashi Y. (2006). Automatic synthesis of 16*α*-[18F]fluoro-17*β*-estradiol using a cassette-type [18F]fluorodeoxyglucose synthesizer. *Nuclear Medicine and Biology*.

[19] Nosova Y. N., Foteeva L. S., Zenin I. V. (2017). Enhancing the Cytotoxic Activity of Anticancer PtIV Complexes by Introduction of Lonidamine as an Axial Ligand. *European Journal of Inorganic Chemistry*.

[20] Dong C., Yang S., Shi J. (2016). SPECT/NIRF Dual Modality Imaging for Detection of Intraperitoneal Colon Tumor with an Avidin/Biotin Pretargeting System. *Scientific Reports*.

