# Supplementary material for: One-Step 18F-Labeling of Estradiol Derivative for PET Imaging of Breast Cancer
Source: Contrast Media Mol Imaging. 2018 Mar 5;2018:5362329. doi: 10.1155/2018/5362329 (PMC5859795; doi:10.1155/2018/5362329)
Supplement: Supplementary Materials — Supplementary date available: ESI-MS and NMR spectra. Figure S1: ESI-MS of compound AmBF3-TEG-ES. Figure S2: 1H-NMR of compound AmBF3-TEG-ES. Figure S3: 13C-NMR of compound AmBF3-TEG-ES. Figure S4: 19F-NMR of compound AmBF3-TEG-ES. [file 5362329.f1.doc]

***Supplementary data***

**One-step ^18^F-labeling of estradiol derivative for PET imaging of breast cancer**

Hongbo Huang^†^, Ke Li^†^, Gaochao Lv, Guiqing Liu, Xueyu Zhao, Qingzhu Liu, Shanshan Wang, Xi Li, Ling Qiu* and Jianguo Lin*

*Key laboratory of Nuclear medicine, ministry of health, Jiangsu Key laboratory of Molecular Nuclear Medicine,* *Jiangsu Institute of Nuclear Medicine, Wuxi 214063, P.R. China*

^†^These authors contributed equally.

** E-mail address:* [*qiuling@jsinm.org*](mailto:qiuling@jsinm.org) *(L. Qiu),* [*linjianguo@jsinm.org*](mailto:linjianguo@jsinm.org) *(J. Lin).*

**Fig. S1.** ESI-MS of compound **AmBF_3_-TEG-ES**


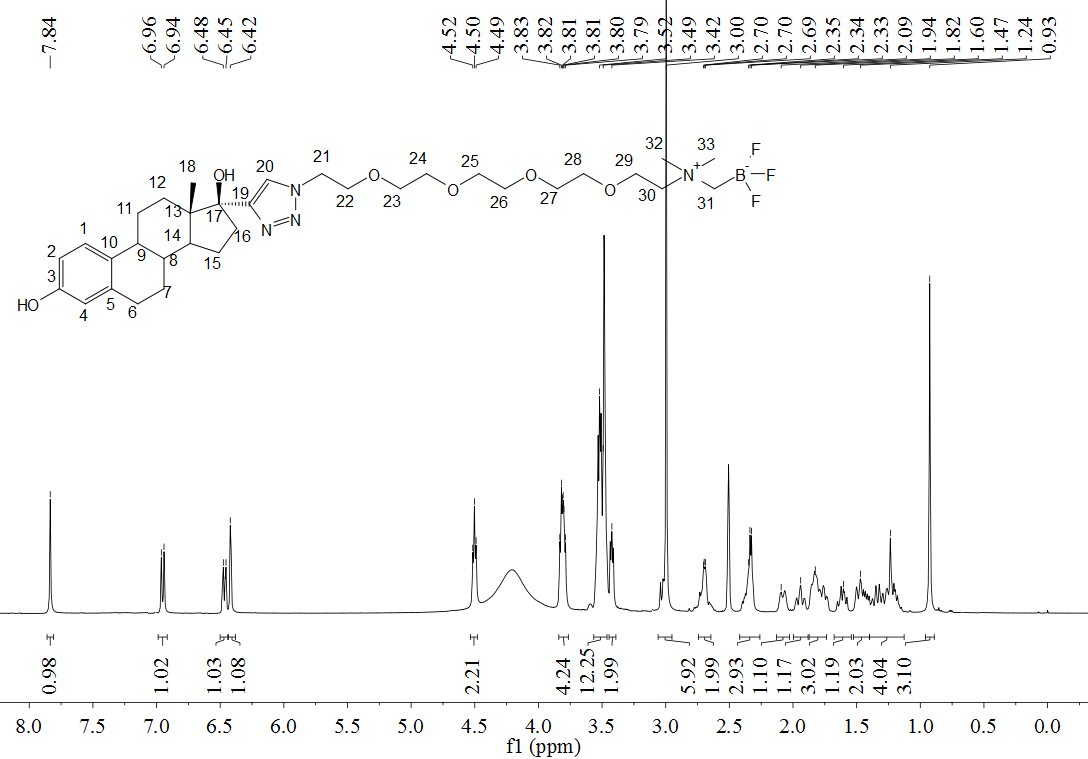


**Fig. S2.** ^1^H-NMR of compound **AmBF_3_-TEG-ES**


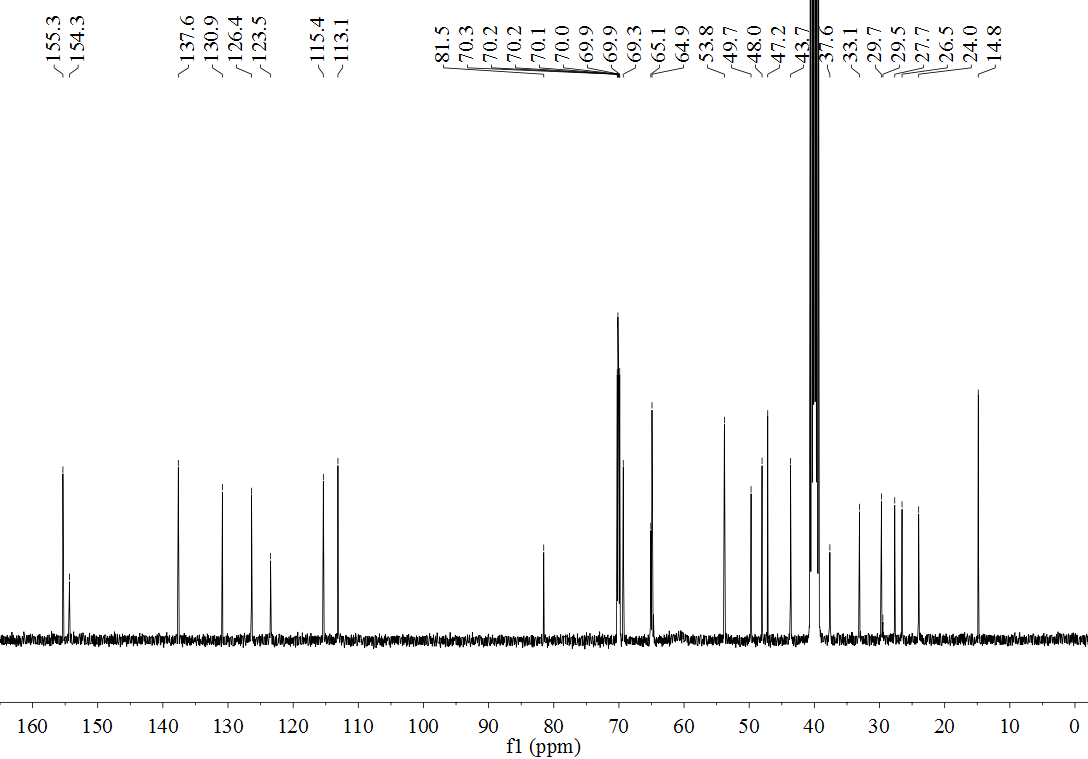


**Fig. S3.** ^13^C-NMR of compound **AmBF_3_-TEG-ES**


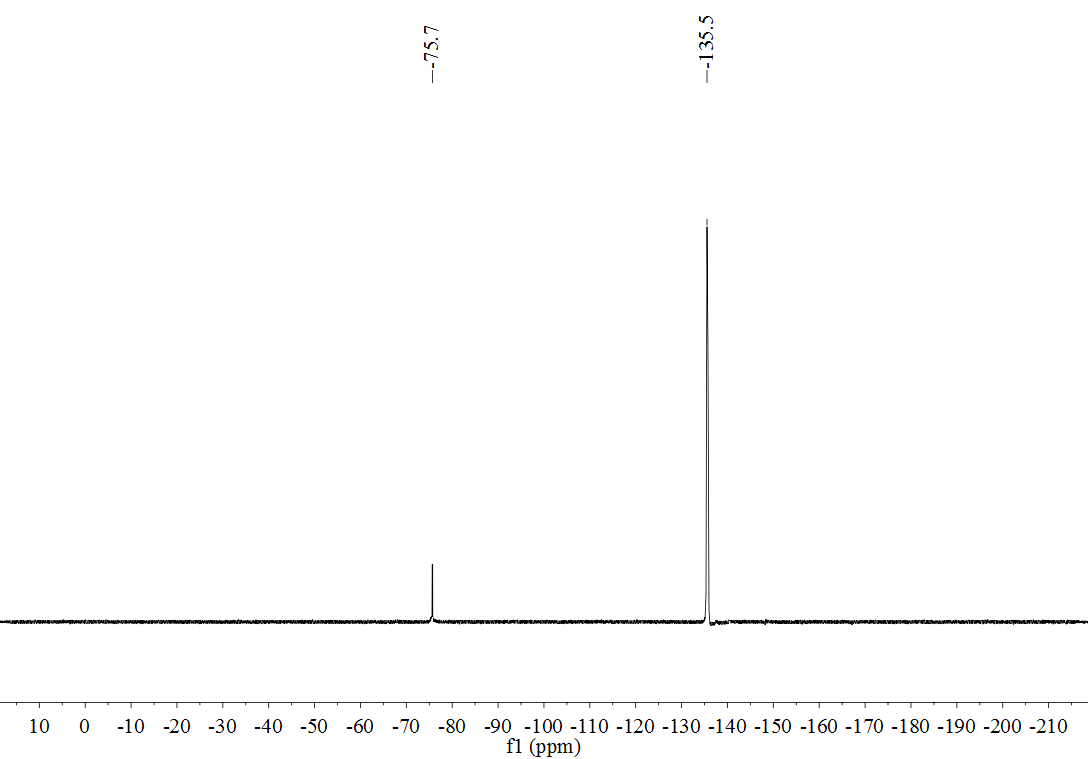


**Fig. S4.** ^19^F-NMR of compound **AmBF_3_-TEG-ES**
